# Bisphosphonate-related osteonecrosis of jaw (BRONJ): an anti-angiogenic side-effect?

**DOI:** 10.1186/1746-1596-7-78

**Published:** 2012-07-06

**Authors:** Eugen B Petcu, Saso Ivanovski, Robert G Wright, Mark Slevin, Rodica I Miroiu, Klara Brinzaniuc

**Affiliations:** 1 Griffith University School of Medicine, Gold Coast Campus, Griffith University, Griffith, QLD 4222, Australia; 2 Doctoral School, University of Medicine and Pharmacy Targu Mures, 38 Gh.Marinescu Street, Targu-Mures, 540000, Romania; 3 Griffith University School of Dentistry and Oral Health, Gold Coast Campus, Griffith University, Griffith, QLD 4222, Australia; 4 Department of Pathology, Gold Coast University Hospital, Nerang Street, Southport, QLD 4215, Australia; 5 Manchester Metropolitan University, Inst for Biomedical Research, John Dalton Building, Oxford Road, Manchester, M1 5GD, UK; 6 Griffith University School of Medicine, Gold Coast Campus, Griffith University, Griffith, QLD 4222, Australia; 7 Department of Anatomy, University of Medicine and Pharmacy Targu Mures, Gh.Marinescu 38, Targu-Mures, 540000, Romania

## Abstract

Bisphosphonates are recommended in the treatment of osteoporosis and some cancers, in which case they prevent the appearance of bone metastasis. The patients taking bisphosphonates are at increased risk of developing bisphosphonate-related osteonecrosis of jaw (BRONJ) which is characterised by the presence of an un-healing wound after dental surgery. BRONJ might represent an anti-angiogenic side effect. However, the real number of patients with BRONJ might be higher than currently recorded. Considering the differential diagnosis which includes various primary and secondary cancers, a correct histopathological diagnosis is very important. The morphological criteria for diagnosis of BRONJ are highlighted in this material.

**Virtual Slides:**

The virtual slide(s) for this article can be found here: http://www.diagnosticpathology.diagnomx.eu/vs/1813972972323288

## 

Bisphosphonates, derivates of pyrophosphates, have been used traditionally to treat hypocalcaemia associated with osteoporosis, multiple myeloma, Paget’s disease and bone metastasis in which case they exert an additional analgesic effect [1]. They bind to the mineralized bone matrix and by acting upon the oscteoclasts inhibit bone resorption. In addition they inhibit formation of new osteoclasts, subsequently creating an unfavourable environment for bone metastasis development [2]. Preclinical and clinical studies suggest that bisphosphonates are able to prevent bone metastasis in a variety of cancers such as breast, lung and prostate. Therefore, we could expect that an increasing number of cancer patients will be taking regularly and for extended periods of time these pharmacological agents [3-5].

Although their value in clinical practice has been proven, the patients taking bisphosphonates are at risk of developing bisphosphonate-related osteonecrosis of jaw BRONJ. By definition, BRONJ is characterised by the presence of an un-healing wound in the maxillofacial region with bone exposure, more than 8 weeks after dental surgery. It seems that patients treated with intravenous bisphosphonates have an increased risk of developing this condition. The incidence is reported to be around 1 in 10,000 patients [6]. In our opinion, considering the large number of patients treated with bisphosphonates, the number of BRONJ cases is largely underestimated and could be significantly higher. A correct histopathological identification of this lesion is of paramount importance since the differential diagnosis includes numerous primary and metastatic tumours.

The biopsy of a BRONJ lesion demonstrates extensive necrosis and inflammation with giant cells (Figure 1 and Figure 2). It is widely accepted that CD105-positive vessels suggest active angiogenesis. However, recent studies conducted on human tissues have shown a significant reduction in CD105-positive vessels in the mucoperiosteal area near the BRONJ zone suggesting inhibition of angiogenesis [7].

**Figure 1:**
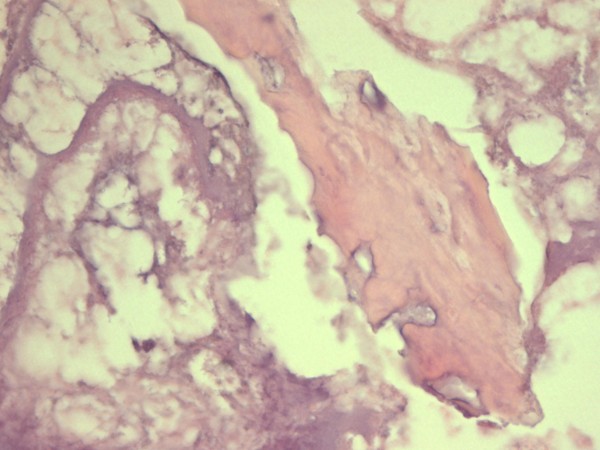
BRONJ: Non-viable bone and extensive osteonecrosis.

**Figure 2:**
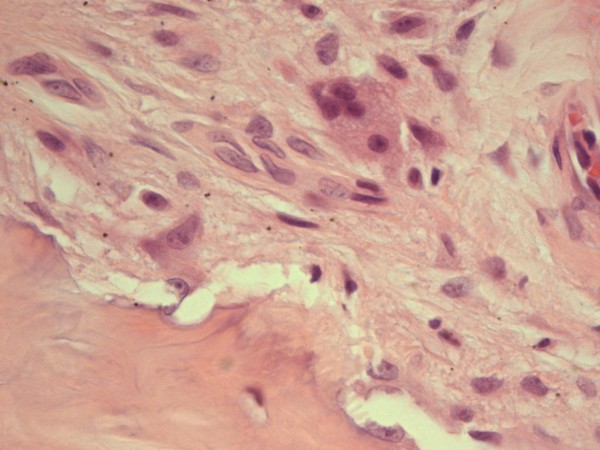
BRONJ: Chronic inflammation with multi-nucleated giant cells.

In vitro and in vivo studies have suggested that bisphosphonates might inhibit IGF-1 induced activation of PI-3 K/Akt/mTOR pathways and have an anti-angiogenic action via inhibition of IGF-1 induced VEGF expression and HIF-1 alpha protein accumulation in MCF-7 cells. [8]. Other studies conducted in mice have revealed that zolendronic acid, a nitrogen-containing bisphosphonate suppresses MMP-9 expression by infiltrating macrophages, decreasing the binding of VEGF to its receptor on angiogenic endothelial cells [9]. In addition, a clinical study conducted in patients with metastatic breast cancer, has revealed that zolendronic acid could exert an anti-angiogenic effect by inducing a transient reduction in VEGF, FGF-2 and MMP-2 [10]. Overall, the above data strongly suggests that bisphosphonates elicit anti-angiogenic effects through a variety of mechanisms that could explain their anti-tumoral action.

In this context, BRONJ represents a frustrating complication for many long-term cancer survivors and osteoporosis patients. Currently, much debate remains on the oetiopathogenesis and management of this condition. We do not know why this lesion appears only in the jaw and maxillofacial area, although the treatment with bisphosphonates is systemic. Mc Leod et al. (2012) suggest that the high turnover of alveolar bone and exposure of the jaw bone may explain the oetiopathogenesis of this condition [11]. However, for a better understanding of this condition more histomorphometrical studies of the maxillary and jaw region should be conducted in parallel with a thorough evaluation of the anti-angiogenic role of bisphosphonates in human tissues and animal models. In conclusion, BRONJ represents a largely underestimated condition due probably to the fact that not many cases are diagnosed accurately by routine histopathology.

## Competing interest

The authors declare that they have no competing interest.

## Authors’ contributions

EBP: drafted the manuscript, provided histopathological material, took digital pictures, SI: helped drafting the manuscript, provided clinical background and interpretation, RGW: provided histopathological evaluation, took digital pictures, MS: provided histopathological research information, helped drafting the manuscript., RIM: helped drafting the manuscript including the tables, provided clinical information, took digital pictures, KB: helped drafting the manuscript, provided basic research information and histopathological evaluation. All authors read and approved the final manuscript.
